# B cell-T cell interplay in immune regulation: A focus on follicular regulatory T and regulatory B cell functions

**DOI:** 10.3389/fcell.2022.991840

**Published:** 2022-09-23

**Authors:** Diaoyi Tan, Wei Yin, Fei Guan, Wanjiang Zeng, Pamela Lee, Fabio Candotti, Louisa K James, Niels Olsen Saraiva Camara, S.M. Mansour Haeryfar, Yan Chen, Kamel Benlagha, Lewis Zhichang Shi, Jiahui Lei, Quan Gong, Zheng Liu, Chaohong Liu

**Affiliations:** ^1^ Department of Pathogen Biology, School of Basic Medicine, Tongji Medical College, Huazhong University of Science Technology, Wuhan, China; ^2^ Department of Urology, Union Hospital, Tongji Medical College, Huazhong University of Science and Technology, Wuhan, China; ^3^ Wuhan Children’s Hospital, Tongji Medical College, Huazhong University of Science and Technology, Wuhan, China; ^4^ Department of Obstetrics and Gynecology, Tongji Hospital, Tongji Medical College, Huazhong University of Science and Technology, Wuhan, China; ^5^ Department of Paediatrics and Adolescent Medicine, Li Ka Shing Faculty of Medicine, The University of Hong Kong, Hong Kong, China; ^6^ Division of Immunology and Allergy, Lausanne University Hospital and University of Lausanne, Lausanne, Switzerland; ^7^ Centre for Immunobiology, Bizard Institute, Queen Mary University of London, London, United Kingdom; ^8^ Department of Immunology, Institute of Biomedical Sciences, University of São Paulo (USP), São Paulo, SP, Brazil; ^9^ Department of Microbiology and Immunology, Western University, London, ON, Canada; ^10^ The Second Department of Pediatrics, Affiliated Hospital of Zunyi Medical University, Zunyi, China; ^11^ Université de Paris, Institut de Recherche Saint-Louis, EMiLy, Paris, France; ^12^ Department of Radiation Oncology University of Alabama at Birmingham School of Medicine (UAB-SOM) UAB Comprehensive Cancer Center, Jinzhou, China; ^13^ Clinical Molecular Immunology Center, School of Medicine, Yangtze University, Jinzhou, China; ^14^ Department of Immunology, School of Medicine, Yangtze University, Jinzhou, China; ^15^ Department of Otolaryngology-Head and Neck Surgery, Tongji Hospital, Tongji Medical College, Huazhong University of Science and Technology, Wuhan, China

**Keywords:** regulatory T cells, B cells, T follicular regulatory cells, regulatory B cells (bregs), humoral responses, germinal center, treg-of-B cells

## Abstract

B cells are the core components of humoral immunity. A mature B cell can serve in multiple capacities, including antibody production, antigen presentation, and regulatory functions. Forkhead box P3 (FoxP3)-expressing regulatory T cells (Tregs) are key players in sustaining immune tolerance and keeping inflammation in check. Mounting evidence suggests complex communications between B cells and Tregs. In this review, we summarize the yin-yang regulatory relationships between B cells and Tregs mainly from the perspectives of T follicular regulatory (Tfr) cells and regulatory B cells (Bregs). We discuss the regulatory effects of Tfr cells on B cell proliferation and the germinal center response. Additionally, we review the indispensable role of B cells in ensuring homeostatic Treg survival and describe the function of Bregs in promoting Treg responses. Finally, we introduce a new subset of Tregs, termed Treg-of-B cells, which are induced by B cells, lake the expression of FoxP3 but still own immunomodulatory effects. In this article, we also enumerate a sequence of research from clinical patients and experimental models to clarify the role of Tfr cells in germinal centers and the role of convention B cells and Bregs to Tregs in the context of different diseases. This review offers an updated overview of immunoregulatory networks and unveils potential targets for therapeutic interventions against cancer, autoimmune diseases and allograft rejection.

## 1 Introduction

**FIGURE 1:**
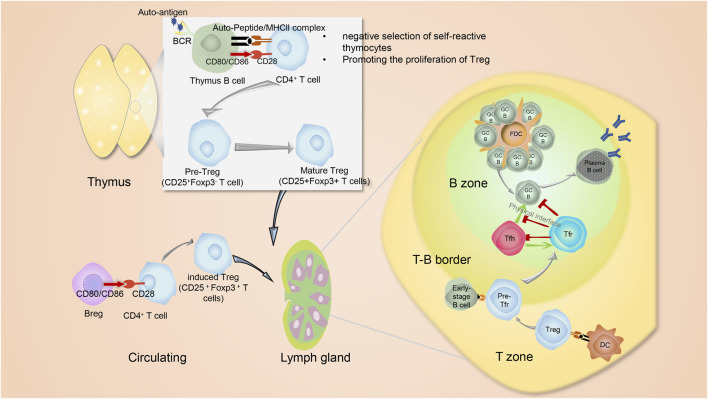
The interplay between B cells and Treg. In addition to thymic mTEC and BM-DCs that act as APCs to promote to produce Tregs, thymus B cells expressing CD80, CD86 and MHC II, also function like APC cells. Their BCRs capture autoantigens. Interaction of TCR-MHC II-peptide and CD28^−^CD80/CD86 between B cells and CD4^+^ T cells lead to the production of thymic CD25^+^Foxp3^-^ pre-Tregs, which then advance into Foxp3+ mature Tregs. In the periphery, naive CD4^+^Foxp3^-^ T cells may interact with immune cells such as Breg and then transform into periphery-derived Tregs. Both thymus-derived Tregs and periphery-derived Tregs would circulate into lymphoid organs, where they will transform into Tfr cells and exert suppressive functions on the humoral immunity via controlling metabolic pathways of both B cells and Tfh cells. The differentiation of Tfr cells occurs in a stepwise manner. The first step involves interactions with dendritic cells (DCs) that initiate imprinting on the Tfr cell development, and the second step is interaction with B cells to strengthen Tfr cell signaling. Tfh, Tfr and GC B cells own complicated and elaborate regulatory interrelationship. Tfh cells deliver positive function to B cells for affinity-matured antibody production. Besides, Tfh cells themselves help drive the formation of Tfr cells. Conversely, Tfr cells can not only act directly on B cells but also suppress Tfh cells to indirectly act on B cells by preventing the co-stimulation and cytokines needed to promote B cell proliferation. Furthermore, Tfr cells can physically disrupt of Tfh-B cell communication. The figures are portrayed with the help from website Servier Medical Art (smart.servier.com).

Regulatory T cells (Tregs) are a unique subpopulation of CD4^+^ T cells, with FoxP3^+^ cells being their most prominent version. *In vivo*, the generation of Tregs occurs in two different ways. The first and major pathway occurs in the thymus where medullary thymic epithelial cells (mTECs) help drive CD4^+^Foxp3^+^ T cells to thymus-derived Tregs (tTregs) by presenting self-antigens (Abbas et al., 2013; Lucas et al., 2016). The autoimmune regulator (AIRE) within mTECs is a transcription factor triggering the expression of tissue-restricted antigens (Lucas et al., 2016). The second pathway operates in the periphery, where naive CD4^+^Foxp3^-^ T cells interact with cognate antigens and transform into periphery-derived Tregs (pTregs) (Abbas et al., 2013) (Figure 1). Although Tregs only make up a small portion of the peripheral T cell pool, they play an indispensable role in maintaining immune homeostasis and self-tolerance. B cells, as another protagonist of this article, originate from bone marrow and are key components of humoral immune responses (Arnon et al., 2013). Mature B cells can be assigned into B-1 B cells and B-2 B cells, and B-2 B cells can be further divided into marginal zone cells (MZ B) and follicular B cells (FO B) (Arnon et al., 2013). A steady stream of research has confirmed the close connection between B cells and Tregs and revealed their complex regulatory relationship. On the one hand, Tregs repress humoral immunity by inhibiting B cells (Bennett et al., 2001; Jang et al., 2011), while on the other hand, not only can B cells regulate the proliferation of naïve CD4^+^ T cells by presenting antigen and co-stimulatory molecules, B cells can also regulate the proliferation and function of Tregs (Ellis and Braley-Mullen, 2015).

Germinal centers (GCs) are specialized structures in B cell follicles (Berek et al., 1991). Upon immunization or (and) infection, GCs transiently form and maintain for weeks to months, which occurs in peripheral lymphoid organs like dLN and Peyer’s patch (Berek et al., 1991). Mature GCs are micro-anatomically divided into the light zone (LZ) and dark zone (DZ) (Chung et al., 2011). In DZ, after several rounds of cellular division, the FO B cells go through somatic hypermutation. Upon receiving stimulus, the B cells migrate from the DZ to the LZ to go through Class-switch recombination (CSR) and selection with the help of follicular dendric cells, Tfh cells and Tfr cells (Rathanaswami et al., 2005; Chung et al., 2011). Finally, these B cells successfully mature into plasma cells with high-affinity antibodies or memory B cells that can be activated once they meet with the same antigens (Rathanaswami et al., 2005). T follicular regulatory (Tfr) cells are a special subgroup of Tregs residing in lymphoid follicles synchronously expressing the molecules belonging to follicular helper T (Tfh) cells and Tregs. Tfr cells have been found to interact with B cells and regulate germinal center responses (Wollenberg et al., 2011). In mice lacking Tfr cells, B cell-associated severe adaptive humoral immune response disorders and autoimmunity disorders have been witnessed (Fu et al., 2018). But the mechanism underlying the regulatory role of Tfr cells to B cells still remains unclear.

Regulatory B cell (Breg) is a broad term to describe B cells with regulatory function (Mauri and Bosma, 2012). Although rare, Bregs still play an essential role in maintaining immune tolerance (Mauri and Bosma, 2012). Among all the regulatory roles of Bregs, their function to promote the expansion of Treg is eye-catching (Wang et al., 2014; Lu et al., 2017; Tarique et al., 2018; Gao et al., 2019). Bregs have been widely identified as key regulators of autoimmunity, tumors and infection (Iwata et al., 2011; Mohammed et al., 2013; Wang et al., 2014; Woo et al., 2014; Wang et al., 2020a), and a series of reports verified that the peripheral blood of patients with diseases contained Bregs, suggesting clinical significance and possible therapeutic targets (Iwata et al., 2011; Mohammed et al., 2013; Wang et al., 2014; Woo et al., 2014; Wang et al., 2020a).

Furthermore, a special subset of Tregs without the expression of FoxP3 but still owning regulatory features has been identified (Xie et al., 2015). Since they are induced by Naïve B cells, they are also termed Treg-of-B cells. The Treg-of-B cells exert their function mainly through cell:cell contact manner and shelter mice from various immune disorders (Chien and Chiang, 2017).

Here in this article, we focus on the interplay between B cells and Tregs. Particularly, we summarize the influences of Tfr cells on B cells in GC B cells and germinal center responses, introduce Bregs and their interaction with Tregs, and review special CD4^+^ Foxp3^-^ Treg-of-B cells (Xie et al., 2015). Besides, we list a series of research from clinical patients and experimental models to illustrate the functions of Tfr cells in diseases and elucidate the regulation of Bregs to Tregs in the context of different diseases, trying to unveil potential therapeutic targets to help the patients in need.

## 2 Suppressive effects of tregs on B cell and humoral immunity

### 2.1 Suppression of B cells by Tregs

B cells can serve two major functions in their capacities as antigen-presenting cells (APCs) and antibody-secreting cells, both of which can be suppressed by Tregs. To this end, early studies demonstrated that Tregs can suppress humoral immune responses both in humans and mice (Bennett et al., 2001; Jang et al., 2011). Treg deficiency is directly correlated with an increased IgE response to food antigens and allergens in mice (Lin et al., 2005). Patients with polyendocrinopathy enteropathy X-linked syndrome (IPEX), an immune dysregulation syndrome which was found to have mutations in *FOXP3*, were associated with high-titers of class-switched autoantibodies (Bennett et al., 2001). In addition, Tregs can suppress the APC function of B cells, as B cells from Treg-deficient mice induce more CD43, Ki-67 and granzyme B from CD8^+^ T cells than B cells from Treg-sufficient mice (Walker and Sansom, 2011; Moore et al., 2019).

### 2.2 The mechanisms underlying suppression of B cells by Tregs

Molecules like Cytotoxic T lymphocyte antigen-4(CTLA-4), transforming growth factor-beta (TGF-β), Fas Ligand (FasL), perforin, granzyme B and Programmed Cell Death Protein 1(PD-1) involve in suppressing B cells by Treg. CTLA-4 is a critical inhibitory molecule that binds to CD86 and CD80 accompanied by higher affinities than CD28. CTLA-4 has cell-intrinsic functions, but on Tregs, it functions in a cell-extrinsic fashion by outcompeting CD28 to deny the availability of CD80 and CD86 for co-stimulation of conventional T cells (Walker and Sansom, 2011). Tekguc et al. demonstrated that CTLA-4 within Tregs would deplete the CD80/CD86 expression in activated B cells through extracting them *via* CTLA-4-dependent trogocytosis (Tekguc et al.). Abatacept is a recombinant protein of CTLA-4 that binds to CD86 and CD80 without inducing downstream signaling in human B cells (Lorenzetti et al., 2019). In patients with Rheumatoid Arthritis (RA), the abatacept treatment leads to reduced plasmablast counts, decreased frequency of self-reactive memory B cells and curtailed serum lgG (Lorenzetti et al., 2019), suggesting that CTLA-4 help Tregs repress B cells.

TGF-β is another inhibitory molecule and is widely secreted by various cell types, while glycoprotein-A repetitions predominant (GARP), type I transmembrane cell surface docking receptors interacting with latent TGF-β to modulate its activation, is especially expressed in Tregs (Tran et al., 2009). TGF-β exerts inhibitory functions on both human and murine B cells and suppresses IgG class switching, but surprisedly promotes the production of lgA (Kehrl et al., 1986; Kehrl et al., 1991). The promotion of IgA by TGF-β is due to activated Runx3 and Smads binding to tandem-repeat elements of α-germline transcript to induce transcription (Park et al., 2001). Additionally, Treg-secreting suppressive molecules, such as lL-10 and IL-35 modulate the proliferation and activation of B cells (Deaglio et al., 2007; Yang et al., 2020).

Beyond the suppressive molecules describe above, cytotoxic molecules like FasL, perforin and granzyme B enforce Treg-mediated suppression of B cells. Rapetti et al. reported that in healthy subjects, suppressing B cells by Tregs required FasL (Rapetti et al., 2015). The expression of Fas in B cells decreased following the interaction with Tregs. The decrease reflects its internalization through an endosomal pathway (Eramo et al., 2004) and the internalization is the prerequisite for apoptosis (Algeciras-Schimnich et al., 2002). Apart from FasL, Zhao et al. revealed that antigen-presenting B cells were killed by Tregs in perforin- and granzyme B-dependent manner (Zhao et al., 2006) and bystander B cells were not affected by Tregs (Zhao et al., 2006).

Recent studies implied that the interaction of PD-1 and PD-L1 could also play a role in Treg-induced B cell apoptosis (Okamura et al., 2015; Gotot et al., 2018). Antigen-specific PD-L1^+^ pTregs, characterized by the absence of Helios and Neuropilin-1, reside in the autoantigen-draining lymph nodes. Upon encountering autoantigen-specific B cells, these Tregs induce the expression of PD-L1 in B cells and suppress the auto-reactive B cells through PD-1-PD-L1 signaling-induced apoptosis (Gotot et al., 2018).

## 3 The introduction of follicular regulatory T cells and its interaction with B cells

### 3.1 Phenotype of follicular regulatory T cells

Tfr cells are a subgroup of Tregs residing in the follicles and being identified as CXCR5^hi^ PD-1^hi^ CD25^low^ Foxp3^+^ Bcl-6^hi^ Blimp1^low^ T cells (Botta et al., 2017; Ritvo et al., 2017). They synchronously express molecules belonging to Tfh cells and Tregs (Wollenberg et al., 2011; Sayin et al., 2018). The molecules related to Tregs (Sayin et al., 2018) are FoxP3, Glucocorticoid-induced tumor necrosis factor receptor-related protein (GITR), PR domain zinc finger protein 1(Prdm1), and CTLA-4. On the other hand, Tfr cells resemble Tfh cells phenotypically in many ways, including their expression of B-cell lymphoma **6** (Bcl-6), inducible Co-Stimulator (ICOS) and C-X-C motif chemokine receptor 5(CXCR5). However, Tfr cells do not express cytokines like interleukin-21 and interleukin-4. Whether Tfr cells are closer to Tfh cells or Tregs, lineage-wise is under debate. On the one hand, in Tfr cells, Bcl-6 decides the transcriptome of Tfr cells more efficiently than Foxp3. Besides, both Tfr cells and Tfh cells lack the expression of CD25, while the expression of CD25 in Tregs is sufficient. On the other hand, Tfr cells approximate to Tregs functionally. They both exert inhibitory effects to prevent exaggerated immune responses, and the TCR repertoire of Tfr cells is more similar to Tregs (Maceiras et al., 2017). Moreover, the expression of Bcl-6 in Tfr cells is far less than that in Tfh cells.

### 3.2 The differentiation of follicular regulatory T cells

Tfr cells are mostly derived from natural Tregs outside the thymus (Wollenberg et al., 2011). Additionally, Tfr cells can also be induced from naïve CD4^+^ T cells when the conditions induce naïve CD4^+^ T cells expressing Foxp3 (Nian et al., 2021). The differentiation of Tfr cells occurs in a stepwise manner. Firstly, Tregs interact with dendritic cells (DCs) to initiate imprinting on the Tfr cell development. Then, precursor Tfr cells migrate toward the B-cell zone, where they interact with B cells to generate fully functional effector Tfr cells (Figure 1) (Sage et al., 2014a). Apart from DCs and B cells, Tfh cells also help drive the formation of Tfr cells (Figure 1) (Essig et al., 2017).

**FIGURE 2:**
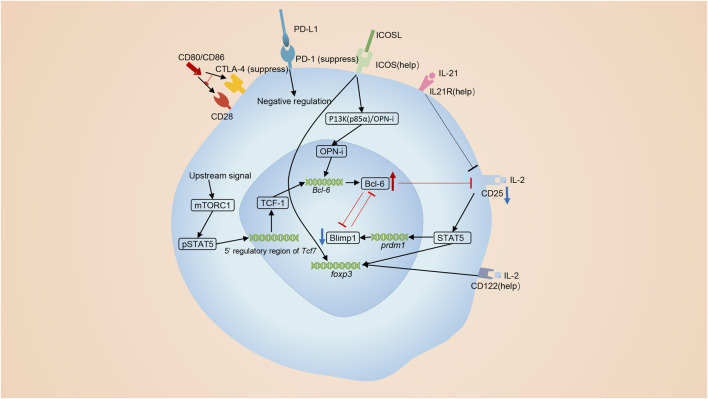
The differentiation of Tfr cells. Tfr cells are primarily derived from Tregs. ICOS signaling promotes the differentiation of Tfr cells. The p85α-OPN-i axis requires ICOS for the Bcl-6-dependent differentiation of Tfr cells. The interactions of CD28 on Tregs and CD80 on B cells is also required for optimal differentiation of Tfr cells. MTORC1 signaling elevates TCF1 to induce the differentiation of Tfr cells. lL-21 also has a positive effect on the number of Tfr cells. On the contrary, co-inhibitory molecules, CTLA4 and PD-1, negatively regulate the differentiation of Tfr cells. Bcl-6 and Blimp-1 are a pair of opposites. IL-2 signaling activates STAT5 and induces the expression of Blimp-1 while Bcl-6 restricts the expression of the receptor of IL-2, CD25.

Molecules like ICOS, CD28, and mTORC1 positively promote the differentiation of Tfr **(** Figure 2 **)**. ICOS exerts indispensable roles in the development of Tfr cells. Activation of ICOS facilitates the interaction of p85α with intracellular osteopontin (OPN-i). Then, OPN-i translocates to the nucleus and interacts with Bcl-6 to interface ubiquitin-dependent degradation of Bcl-6 (Leavenworth et al., 2015). Mammalian target of rapamycin complex 1 (mTORC1)-related signals also exert essential roles in the differentiation of Tfr cells (Xu et al., 2017a). Blocking mTORC1 signaling using rapamycin inhibits the initial conversion of Tregs to Tfr cells, and overexpression of Bcl6 or TCF1 can reverse this poorly differentiated state in Raptor-deficient Tregs (Xu et al., 2017a). Besides, the interaction between CD28 on Tfr cells and CD80 on B cells is also demanded for optimal differentiation of Tfr cells as *Cd*28^-/-^ mice lack Tfr cells (Linterman et al., 2011).

On the contrary, signalings from co-inhibitory molecules CTLA4 and PD-1 reversely regulate the differentiation of Tfr cells (Miyazaki et al., 2014; Botta et al., 2017; Jandl et al., 2017) (Figure 2). PD-L1 on B cells and DCs mediates inhibitory signaling to Tfr cells and suppresses the early differentiated stage of Tfr cells from Tregs. Interference of PD-1-PD-L1 interaction increases the number of Tfr cells (Zeng et al., 2020). CTLA-4 also harms the differentiation of Tfr cells, as Tfr cells expand in blood and lymph nodes when depletion of CTLA-4 is induced 3 days before immunization (Sage et al., 2014b).

IL-21, cytokines created by Tfh cells, Th17 and NK cells, profoundly affects the function of Tfh cells, plasmablasts, and plasma cells in GCs (Søndergaard and Skak, 2009; Spolski and Leonard, 2010; Alahgholi-Hajibehzad et al., 2017). However, the relationship between IL-21 and Tfr cells is not clear. So far, we knew that the production of IL-21 was inhibited by Tfr cells and IL-21 played an essential role in negatively modulating Tregs (Fabrizi et al., 2014), but the impact of IL-21 on the development Tfr cells remained controversial. Research made by Peter T Sage et al. exhibited that IL-21 suppressed the cell cycling of Tfr cells using *in vitro* experiments (Sage et al., 2016). The development of Tfr cells involves a transition from CD25^+^ state to CD25^−^ state (Botta et al., 2017) and CD25^+^ Tregs localized in the T cell zone act as a precursor population and regulate earlier stages of B cell responses (Botta et al., 2017). But Peter T Sage et al. sorted CD4^+^ICOS^+^CXCR5^+^Foxp3^+^CD19^−^ or CD4^+^ICOS^+^CXCR5^+^GITR^+^CD19^−^ as Tfr cells, ignoring that cells with different levels of CD25 represented different cells subtypes (Sage et al., 2016). Thus, in my opinion, their conclusion can’t represent the real effect of IL-21 on Tfr cells. Jandl et al. demonstrated that lack of IL-21 augmented the percentage and absolute number of FoxP3^+^ Treg and CD25^+^CXCR5^+^ precursor Tfr cells, but had no influence to CD25^−^ mature Tfr cells. They also pointed out that IL-21 signaling was able to reduce the expression of CD25 on Tfr cells (Jandl et al., 2017). Thus, we think that IL-21 exerts a unique function in the development of Tfr cells. It does restrict the proliferation of precursor Tfr cells, but meanwhile sustain the development of mature Tfr cells (Figure 2). Referring to that IL-21 exerts its function *via* JAK/STAT, MAPK and PI3K pathway (Habib et al., 2002; Spolski and Leonard, 2008), we infer that the influence of IL-21 on Tfr cells is probably in a Tfr cell-intrinsic manner, and notably, this intracellular function is exclusively for Tfr cells, but not for non-follicular Treg (Jandl et al., 2017).

Bcl-6 and Blimp-1 exert essential but opposing functions for the development of Tfr cells (Johnston et al., 2009). Bcl-6 is significantly upregulated in Tfr cells while Blimp-1 is significantly down-regulated (Botta et al., 2017; Ritvo et al., 2017). At the apex of the infection, high concentrations of lL-2 preclude Tfr cell maturation by augmenting Blimp-1 expression and suppressing Bcl-6 expression (Botta et al., 2017) (Figure 2). However, once the inflammatory response is relieved and IL-2 concentrations drop, some precursor Tfr cells reduce CD25 expression and increase Bcl-6 expression, promoting the transformation into Tfr cells (Botta et al., 2017; Wing et al., 2017). Blimp-1 is known as an antagonist of Bcl-6 and can also be suppressed by Bcl-6 (Cimmino et al., 2008). Bcl-6 restricts the expression of CD25 (Jandl et al., 2017) and the low IL-2-CD25 signaling in Tfr cells downregulate Blimp-1 in a STAT5-dependent manner (Ballesteros-Tato et al., 2012; Johnston et al., 2012) (Figure 2). Although Blimp-1 is downregulated in Tfr cells, it is indeed indispensable. Partial reduction of Blimp-1 results in increased numbers of Tfr cells, while Tfr cells with total absence of Blimp-1 loss suppressive function (Shen et al., 2019; Xie et al., 2019).

The decreased CD25 is a hallmark to identify Tfr cells. Mature Tfr cells were characterized as CD25^-^Foxp3^+^ cells located mostly in the center B cell follicle (Chung et al., 2011). As we mentioned above, IL-21 signaling and Bcl-6 are able to downregulate the expression of CD25. Our team previously demonstrated that ubiquitin-specific peptidase 18 (USP18) was able to lower the expression of CD25 on Tregs, but whether USP18 was included in the differentiation of Tfr cells was unknown (Yang et al., 2021). Additionally, IL-2-CD25 signaling promotes the expansion of Tregs through activating phosphorylated STAT5 to bind both the promoter and intron of the *FOXP3* gene (Mao et al., 2019). As Tfr cells mature, the interaction between IL-2 and the CD25 cells gradually decreases. However, Foxp3 expression in mature Tfr cells remains high. This implies that multiple mechanisms function in regulating the expression of Foxp3. Firstly, except CD25, Tfr cells express CD122, the IL-2 receptor β chain presenting in both high- and intermediate-affinity IL-2 receptors (Yuan et al., 2018). Secondly, tonic IL-2/STAT5 signaling may be enough to prevent the downregulation of Foxp3 in Tfr cells (Mao et al., 2019). Thirdly, IL-2 is able to activate Mammalian STE20-like kinase 1 (MST1) and activated Mst1 has the potential to sense and amplify the IL-2-STAT5 pathway in Trges (Shi et al., 2018). Lastly, it has also been reported that IL-7, IL-15 and ICOS are able to participate in the stabilization of Foxp3^+^ cells lacking IL-2/STAT5 signaling (Vang et al., 2008; Gratz et al., 2013; Raynor et al., 2013).

### 3.3 The function of follicular regulatory T cells in the germinal center

Tfr cells can inhibit GC B cells from producing antibodies and adjust class-switch recombination (CSR). When B cells are cultured with Tfh cells alone, the proportion of plasma cells and the amount of IgG are high^74.^ However, after adding Tfr cells to this co-culture, the proportion of plasma cells and the amount of IgG are remarkably reduced (Liang et al., 2020). The elaborate effect of Tfr cells on self-reactive B cells or external antigen-specific B cells is under debate. To be sure, Tfr cells can modulate the responses of autoantibodies-secreting B cells. In circulating blood from systemic lupus erythematosus (SLE) patients, the proportion of Tfr cells declined and the proportion of Tfh elevated (Xu et al., 2017b), and the aberrant Tfh/Tfr ratios is positively associated with the level of anti-double-stranded DNA (dsDNA) antibody in serum from SLE patients (Xu et al., 2017b). Nevertheless, the relationship between Tfr cells and adaptive humoral immune responses is controversial. Botta et al. demonstrated that depletion of Tfr cells only advanced the accumulation of self-reactive B cells, but did not remarkably impact influenza-specific B cells (Botta et al., 2017), which was consistent with the notion that the TCR repertoire of Tfr cells more likely interact with self-antigens (Maceiras et al., 2017). However, other experiments revealed that Tfr cells could exert regulatory function on B cell responses after immunization with soluble antigens (Aloulou et al., 2016). Here, we are inclined to favor the latter opinion.

Firstly, we discuss the relationship between Tfr cells and adaptive humoral immune responses. Both Wu et al. and Fu et al. confirmed that Tfr cells regulated adaptive humoral immune responses by using Bcl6^fl/fl^ Foxp3^Cre^ mouse model (Bcl6FC) to reduce the number of Tfr cells and impair the localization of Tfr cells in GCs (Wu et al., 2016; Fu et al., 2018). Interestingly, the two teams found a common fact that the Bcl6FC mice showed no alteration in the proportion and number of Tfh cells and GC B cells after immunization in dLNs and spleen in comparison with control mice (Wu et al., 2016; Fu et al., 2018), which demonstrates that the effect of Tfr cells on the number of GC B cells is limited, and it is more likely for Tfr cells to achieve a regulatory role in adaptive humoral immune responses by affecting the function of GC B cells. other research also reported that Tfr cells permitted activation of B cells but inhibited effector responses, like CSR and antibody production (Sage et al., 2016). Clement developed different CXCR5^IRES−LoxP-STOP-LoxP-DTR^ Foxp3^IRES−CreYFP^ mouse models (TFR–DTR mouse strain), in which Tfr cells were selectively depleted following administration of diphtheria toxin (Clement et al., 2019). Compared with Bcl6FC mice, Tfr-DTR mice mimicked the normal immune responses more efficiently. From Tfr-DTR models, Clement et al. found that Tfr cells regulated the development of GC B cells and the generation of antigen-specific antibodies at an early stage before the development of the GCs, whereas their effects were negligible once the GCs was initiated. However, Wing et al. suggested that the very early stage of the GC response was controlled by Tregs which resided in the T-cell zone, rather than by precursor Tfr cells at the T-B border or mature Tfr cells in the follicle (Wing et al., 2017). Interference at this phase would augment a mass of GC B and Tfh cells and preserve the antigen specificity of the response, as well as inducing the generation of both extraneous- and auto-reactive antibodies. And the later stage of the GC response was modulated by mature Tfr cells in follicles (Wing et al., 2020). The precise timing when Tfr cells exert function needs further identification.

As for the role of Tfr cell in secondary immune response, different immunization models draw different conclusions. Fu et al. found that Tfr cells limited immune responses to persistent antigens since memory B cell differentiation and antibody affinity maturation were magnified in Bcl6FC (Fu et al., 2018). However, Clement et al. demonstrated that Tfr cells helped to augment secondary immune response, as the affinity of antigen-specific antibodies was improved during the memory response in TFR–DTR mouse (Clement et al., 2019). Likewise, in Wu et al.‘s research, When the Bcl6FC mouse model was treated with HIV-1 gp120 vaccine in a “prime-boost” manner, IgG antibodies showed a remarkably decreased affinity to antigen, but the concentration of anti-gp120 IgG was normal (Wu et al., 2016). Thus, in vaccine-challenged Bcl6FC mouse models, Tfr cells behaved favorably in retaining the affinity of anti-specific IgG antibodies (Wu et al., 2016). Probably, these differences relate to the models used in their respective studies. In summary, we think that Tfr cells can suppress the formation of antigen-specific antibody-secreting cells (ASCs) and regulate the secondary immune response.

Tfr cells are also closely associated with autoimmunity. In general, Tfr cells damped the occurrence of self-reactive B cells and restrict the antibody responses to self-antigens. 6-week-old Bcl6FC mice without any additional treatment had the same numbers of Tfh cells and GC B cells as in the WT mice (Fu et al., 2018). However, at 12 weeks, the Bcl6FC mice had increased numbers of Tfh cells and GC B cells in the spleen, MLNs and pLNs (Fu et al., 2018). Furthermore, as Bcl6FC mice aged, inflammatory cells penetrated organs and high concentrations of serum autoreactive antibodies were detected (Fu et al., 2018). Cytokines produced by the CD4^+^ T cells, such as IL-4 and IFN-γ together with IL-17A, did not change significantly between 30-week-old Bcl6FC and control mice, suggesting that the deficiency of Tfr cells directly acts on B cells with selective effects on GC reactions (Wu et al., 2016). The existence of Foxp3 CD25^+^ precursor Tfr cells accounts for the age-related differences seen in Bcl6FC mice. Precursor Tfr cells reside at the T-B border and function to constrain the development and activity of Tfh cells and B cells in LNs (Sayin et al., 2018). Thus, these cells likely compensate for the lack of Tfr cells in Bcl6FC mice at the early stage. Likewise, in TFR–DTR mice treated with diphtheria toxin before immunization, self-reactive IgG and IgE were detected compared with control mice (Clement et al., 2019).

### 3.4 Molecular mechanisms included in the modulation of follicular regulatory T cells

Next, we delve into the mechanism of Tfr cells regulating B cells. Roughly speaking, Tfr cells function directly on B cells and suppress Tfh cells to indirectly act on B cells by preventing the co-stimulation and cytokines needed to promote B cell proliferation (Figure 1) (Chung et al., 2011). Here in this section, we mainly discuss how Tfr cells directly act on B cells.

**FIGURE 3:**
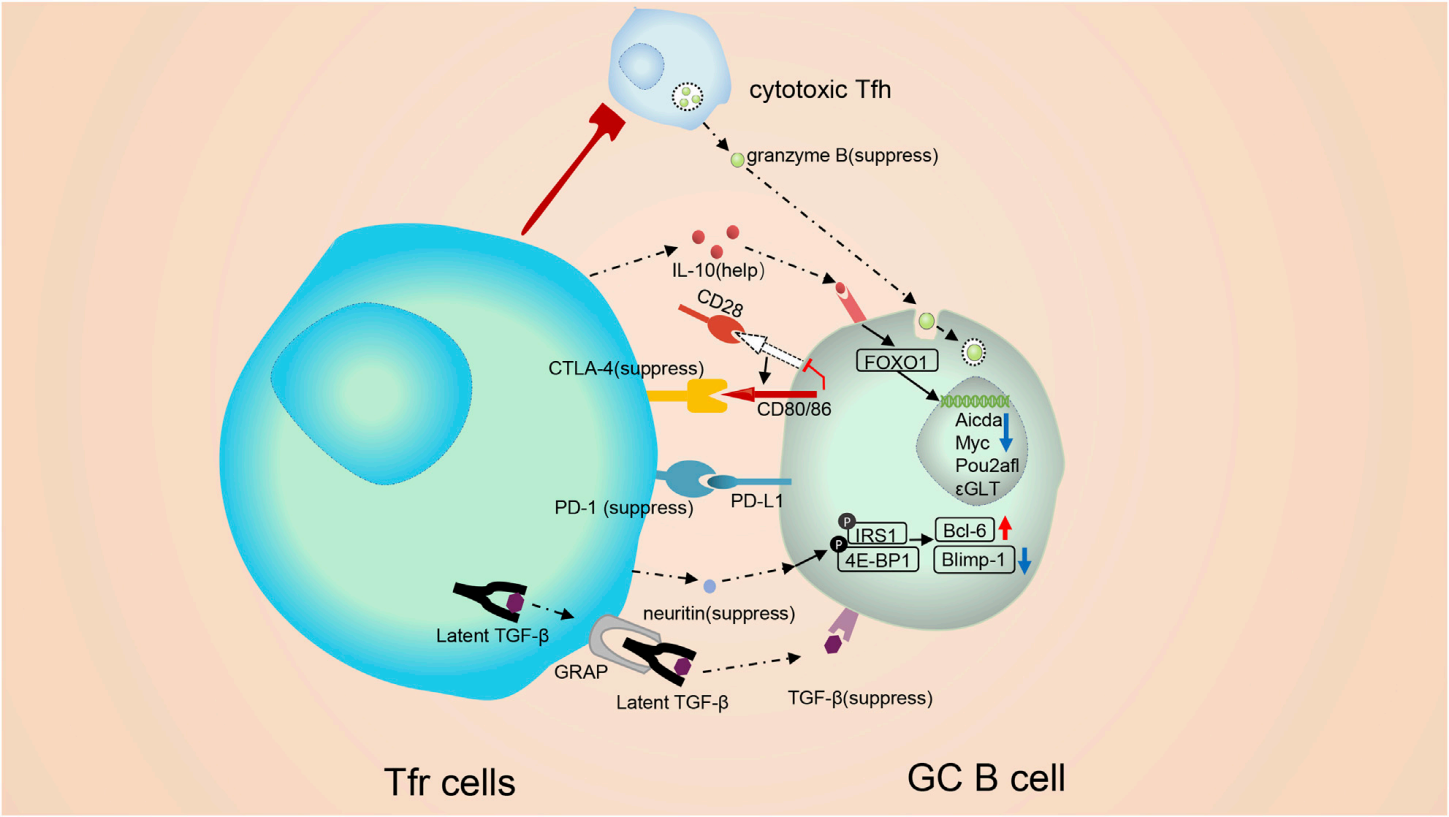
Molecular mechanisms involved in the modulation of follicular regulatory T cells. Tfr cells monitor B cells both through cell: cell contact and cytokines. Tfr cells can alter vital signal pathway in B cells, such as mTOR and Myc pathways, and trigger epigenetic modification. CTLA-4 mediates the suppressive function of Tfr cells without altering the expression of B7-1 or B7-2. Besides, Tfr cells may regulate GC B cells in a TGF-β-dependent way. On the contrary, PD-1 can inhibit the repressive function of Tfr cells. PD-1 reduction in Tfr cells triggers a strong TCR signal, leading to a substantial stop signal which prolongs the communication of B cell with Tfr cells. IL-10 form Tfr cells exerts positive functions on the GC response. IL-10 can promote the production and nuclear translocation of FOXO1. Elevated FOXO1 activate the transcription of multiple genes. cytotoxic Tfh cells secrete abundant granzyme B, which could demolish GC B cells. Tfr cells are capable of suppressing cytotoxic Tfh cells and sustaining the homeostasis of the GC response. Neuritin from Tfr cells is absorbed by B cells and then triggers phosphorylation of proteins like lRS1 and 4E-BP1, augments the expression of Bcl-6 and downregulates the expression of Blimp-1 on GC B cells to suppress the maturation of GC B cells, prevent the formation of self-reactive B cells and prevent class switch recombination (CSR) to IgE.

CTLA-4, TGF-β and neuritin are essential for Tfr cells to suppress GC B cells. Tfr cells damped antibody responses partly in a CTLA- 4-dependent manner (Sage et al., 2014b) (Figure 3). In mice lacking CTLA-4 in Tregs, Wing et al. observed expanded numbers of plasma cells and memory B cells, and raised serum IgE (Wing et al., 2014). Tregs can exert suppressive functions outside GCs by outcompeting CD28 and downregulating CD86 expression in CTLA-4-dependent manners. However, in GCs, CTLA-4 decides the suppressive functions of Tfr without altering the expression of CD80 or CD86 (Sage et al., 2014b). Tfr cells regulate the antibody responses in TGF-β-dependent ways, too. (Sayin et al., 2018) (Figure 3). It was reported that the TGF-β signaling was able to interrupt Tfh cell accumulation in follicles and repress autoantibody production (McCarron and Marie, 2014). Besides, the TGF-β signaling induces the IgA class switching (Cong et al., 2009). Neuritin, a highly conserved neuropeptide, is also identified as a regulator of Tfr cell-mediate inhibitory effect (Gonzalez-Figueroa et al., 2021). Tfr cells are able to express neuritin, which is then absorbed by B cells and then triggers phosphorylation of numerous proteins like lRS1 and 4E-BP1, augments the generation of Bcl-6 and downregulates the generation of Blimp-1 on GC B cells to suppress the maturation of GC B cells, prevent the development of self-reactive B cells and prevent the output of ε germ line transcripts (ε-GLTs) required for CSR to IgE (Gonzalez-Figueroa et al., 2021). IRS1 phosphorylation is needed for the maturation of GC B cells and CSR against IgE (Keegan et al., 2018). 4E-BP1 phosphorylation, the downstream of mTOR signal, is essential for B cells to migrate to the dark zone to undergo somatic mutation (Ersching et al., 2017).

In addition to inhibiting the GC response, Tfr cells can also exert positive regulation on the GC response. The optimal development of GC B cells requires IL-10 from Tfr cells (Laidlaw et al., 2017) (Figure 3). Tfr cell-derived IL-10 promotes the production and nuclear translocation of FOXO1 in B cells (Laidlaw et al., 2017). Elevated FOXO1 activates the transcription of multiple genes like CXCR4, which is needed for GC B cells to migrate into the dark zone (Sander et al., 2015). It is worth noting that Treg-derived IL-10 also initiates STAT3 (Laidlaw et al., 2017) to modulate the development of GC B cells. Our team also has confirmed the importance of STAT3 for GC B cells, as in Mb1^Cre^stat3^flox/flox^ mice, a model with STAT3 specifically deficient in B cells, GC B cells were decreased (Du et al., 2021). Depletion of STAT3 was able to upregulate miRNA146A and then promoted 14-3-3σ, a central hub protein, to upregulate the class-switch DNA recombination (CSR) to IgE in B cells (Du et al., 2021).

Tfr cells own some self-adjusting mechanisms to regulate their inhibitory function of themselves. Apart from inhibiting Tfr cell proliferation, PD-1 signaling can also repress the repressive function of Tfr cells (Figure 3) (Sage et al., 2013). Data showed that Tfr cells from Pdcd1^-/-^ mice controlled antibody production more effectively both *in vitro* and *in vivo* (Sage et al., 2013). PD-1 reduction in Tfr cells triggers strong TCR signals, leading to a substantial-stop signal which prolongs the communication of B cells with Tfr cells (Fife et al., 2009).

Thus, Tfr cells have complex roles in modulating the GC responses. They alter the expression of specific molecules and change pathways associated with the metabolism of GC B cells but maintain their transcriptional signatures (Sage et al., 2016). GC B cells in the LZ elevate Myc expression *via* interacting with antigens, trigger the cell cycle and then move to the dark zone (DZ) to complete mitosis (Calado et al., 2012). *In vitro*, Tfr cells can alter the mTOR and Myc pathways in B cells (Sage et al., 2016). Additionally, Sage et al. used ATAC-sequencing to assess chromatin accessibility of GC B cells suppressed by Tfr cells and found the Aicda, Myc and Pou2afl were inaccessible, suggesting epigenetic modification (Sage et al., 2016). These modification affectes the transcription of genes encoding the upstream metabolic regulators of B cells.

Tfr cells are not the only Tregs in follicles. Canete et al. described a novel cell population in follicles named CD25^+^ T_f_ cells, which lacked the expression of FoxP3 and were identified as CXCR5^hi^PD-1^hi^CD25^+^CD127^-^ cells (Cañete et al., 2019). Though the deficiency of FoxP3, CD25^+^ T_f_ cells are still viewed as a subpopulation of Treg, since these cells express CTLA-4 and lack the secretion of IL-2 (Cañete et al., 2019). CD25^+^ T_f_ cells are abundant in tonsils and are the primary source of IL-10 among human Tregs. CD25^+^ T_f_-derived IL-10 can act on GC B cells and inhibit ε-GLTs to dampen CSR to IgE (Cañete et al., 2019). Besides, CD25^+^ T_f_ can also act on Tfh cells in a non-IL-10-dependent fashion to reduce the expression of Bcl-6, CD40L and IL-21 and indirectly limits the development of GC B cells (Cañete et al., 2019).

Next, we briefly introduce some newfound indirect functions of Tfr cells. Tfr cells can modulate Tfh-mediated GC B cell death and physically disrupt Tfh-B cell communication (Figure 1). In Bcl6^fl/fl^Foxp3^Cre^ mice, a new subpopulation of Tfh cells with cytotoxicity was found (Ritvo et al., 2017) (Figure 3). These cells express abundant granzyme B to induce GC B cell death (Ritvo et al., 2017). Thus, Tfr cells may suppress the cytotoxic Tfh cells and maintain GC B cells. Besides, during the inhibition process, Tfr cells physically disrupt the association between Tfh and B cells (Sage et al., 2014a; Sage and Sharpe, 2015).

In GCs, the phenotype of cells is not fixed and Tfh cells may transfer into Tfr cells in a particular micro-environment. And the transformation from Tfh cells to Tfr cells also influences GC B cells. Hao et al. demonstrated that in *ex vivo* co-culture system involving Tfh cells and B cells, additional treatment of IL-2 leaded STAT3 and STAT5 selectively bounding to the loci of FoxP3 and Bcl-6. The repressive histone modification marker H3K27me3 was also inhibited in Tfh cells. Ultimately, the Tfh cells transformed into Tfr cells to exert inhibitory effects on B cells (Hao et al., 2021).

### 3.5 Diseases involved in modulation of follicular regulatory T cells and their effects on germinal centers

Next, we mainly study the roles of Tfr cells in diseases. Tfr cells play essential regulatory functions in autoimmune diseases. In the peripheral circulation of patients with myasthenia gravis (MG), primary biliary cholangitis (PBC) and autoimmune hepatitis (AIH), the population of Tfr cells is significantly increased to confront self-reactive responses, and the number of Tfh cells reduced, and in general, the ratio of Tfh/Tfr cells is dramatically decreased (Wen et al., 2016; Zheng et al., 2017; Liang et al., 2020). In patients with MG, as the patients get severe, more Tfr cells are activated to regulate the self-reactive rsponses (Wen et al., 2016). Moreover, in MG patients, Glucocorticoid (GC) therapy relieves clinical symptoms, and the imbalance of the circulating Tfh/Tfr ratio is restored (Wen et al., 2016).

In AIH patients, levels of Tfr cells are significantly higher, which is positively correlated with serum concentrations of TGF-β and IL-10, and inversely correlated with the frequency of circulating Tfh cells and serum immunoglobulin (Liang et al., 2020). This phenomenon demonstrates that Tfr cells exert an immunoregulatory role in human beings by regulating immunoglobulin production (Liang et al., 2020). In the Systemic lupus erythematosus (SLE) mouse model, Bcl6FC mice develop a strong elevation of auto-reactive IgA titers, which resembles the high titers of IgA anti-dsDNA antibodies in patients with SLE that cause joint abnormalities and kidney injury (Villalta et al., 2013)^.^. Likewise, in the Empty Sella Syndrome (ESS) model, Bcl6FC mice exhibit increased auto-reactive antibodies (Wu et al., 2016). These phenomena support the notion that Tfr cells exert vital roles in controlling the GC responses in autoimmune disease (Wu et al., 2016).

Tfr cells play a central mechanism to suppress the IgE-mediated allergies (Gonzalez-Figueroa et al., 2021). Reduced frequencies of Tfr cells have also been observed in the tonsils and peripheral circulation of patients with allergic rhinitis (Schulten et al., 2018; Yao et al., 2019). *in vitro*, Tfr cells from allergic rhinitis patients retain the potential to suppress the generation of IgA, IgM, IgG, except for IgE (Yao et al., 2019). *In vivo*, Tfr cells control the infiltration of immune cells during allergen sensitization, which modulates antigen-specific IgE responses (Clement et al., 2019). Thus, Tfr cells could potentially be a targeted therapy for allergen immunotherapy, as the increased Tfr cells are positively associated with improved clinical outcomes (Schulten et al., 2018; Yao et al., 2019).

### 3.6 Tregs with chimeric antigen receptor technology

Tregs have essential roles in suppressing immune responses. How to utilize the suppressive properties in treating patients with autoimmune diseases, allergies, graft-versus-host diseases (GvHD) as well as cancers has become a priority in research. Researchers have succeeded in using adoptive transfer of *ex vivo* expanded polyclonal Tregs into subjects to inhibit GvHD after transplantation of allogeneic hematopoietic stem cells (Brunstein et al., 2011; Theil et al., 2015). The therapeutic effect is promising, but the risks of nonspecific immunosuppression are obvious. Chimeric Ag receptor-expressing T (CAR-T) cell therapy has been widely used in treating B-cell lymphoma patients. Based on CAR-T technology, adoptive transfer of CAR Tregs can be an alternative to *ex vivo* expanded polyclonal Tregs.

Yoshimura et al. successfully established a method for producing and culturing CD19-CAR CD45RA + Tregs (Imura et al., 2020). They first enriched and purified naive/resting Tregs (CD45RA^+^ Tregs) and infected them with CD19-CAR retroviruses. Then the infected Tregs were selected out and amplified for 8 days through culturing with human CD19^+^ K562 cells and IL-2l (Imura et al., 2020). CD19-CAR CD45RA^+^ Tregs maintain the expression of Helios, CTLA-4 and IL-10 like regular Treg and can persist for a long time. Abundant surface latency-associated peptides (LAP) and GARP are also expressed in CD19-CAR CD45RA^+^ Tregs. Most importantly, unlike prevailing CAR T cells, CAR Tregs have few cytotoxic activities. *In vitro* experiments show that CD19-CAR Tregs inhibit the generation of antibodies *via* TGF-β signaling (Imura et al., 2020). Furthermore, in the xenogeneic GvHD model, CD19-CAR Tregs repress the generation of antibodies and inhibit the differentiation of mature B cells to plasma cells without harming or killing B cells. Thus, compared to CD8^+^ CAR T cells, CD19-CAR Tregs are safer and can be a new therapeutic method in autoimmunity.

Hemophilia A (HA) patients with therapeutic FVIII treatment eventually generate anti-factor VIII (FVIII) neutralizing antibodies. Zhang et al. developed a modified CAR Tregs to induce specific tolerance and decrease the secretion of neutralizing antibodies (Zhang et al., 2018). They developed a B cell-targeting antibody receptor (BAR) with an extracellular domain comprising immunodominant FVIII C2 or A2^103^. This was done using retroviruses to transduce BAR into Tregs to form BAR-Tregs. Such BAR-Tregs can directly aim at FVIII-specific B cells to prevent anti-FVIII antibody secretion upon FVIII immunization (Zhang et al., 2018).

## 4 Regulating Tregs by B cells

### 4.1 The abundance and function of Tregs differ, in the presence or absence of B Cells

Except that Tregs influence B cells, in turn, studies demonstrates that the number and frequency of Tregs are also affected by B cells. Gene-modified B cell-deficient mice with treatment of dextran sulfate sodium (DSS) showed a decreased number of Tregs and demonstrated more severe colitis, compared with B cell-sufficient mice, and adaptive transfer of B cells into the B cell-deficient mice with colitis fixed the number of Treg (Lund and Randall, 2010; Weber et al., 2010; Wang et al., 2015). The same results can be extracted from B cell-deficient (μMT)mice with Friend Virus infection (Moore et al., 2017). In MOG protein-induced EAE mice with the treatment of CD-20, the depletion of B cells was correlated with the decreased Treg both in peripheral lymphoid organs and within the central nervous system (Lund and Randall, 2010; Weber et al., 2010; Wang et al., 2015). In B-cell-deficient mice with EMT-6 mammary adenocarcinoma cells, the population of Tregs in the spleen, tumor draining lymph nodes, and tumor bed were remarkably declined compared to wild-type mice. However, Yu et al. showed that the absolute number of Tregs in the spleen was higher in mice with SAT plus those treated with anti-CD20 (Yu et al., 2012). In our opinion, Particular autoimmune models and inflammatory circumstances may influence whether Tregs are elevated, declined or unchanged after B cell depletion.

Apart from the impact in quantity, the function and surface molecules of Tregs are also affected by B cells. The suppressive effects of Tregs in mice deficient with B cells is more potent than that of Tregs in B cell-sufficient mice (Ellis and Braley-Mullen, 2015). The capacity of Tregs from anti-CD20 treated mice in suppressing experimental arthritis is greater than Tregs from untreated mice (Hamel et al., 2011; Ellis and Braley-Mullen, 2015). Tregs from B cell-depleted NOD. H-2h4 mice are superior in their capacity to repress spontaneous autoimmune thyroiditis (SAT) compared to Tregs from WT mice (Ellis and Braley-Mullen, 2015). In SLE or mixed cryoglobulinemia vasculitis patients, the functions of Tregs are improved after B cell depletion therapy by rituximab (Lund and Randall, 2010). However, in myelin oligodendrocyte glycoprotein (MOG) -induced EAE mice, Tregs from B cell-depleted mice and wild type have indistinguishable suppressive function (Hoehlig et al., 2012). Part of the reason for this difference is that the formation of EAE induced by MOG peptide does not require B cells, while B cells are essential for the development of SAT (Hoehlig et al., 2012; Ray et al., 2012). Tregs from B cell-depleted mice show significant differences in surface molecules compared with B cell-sufficient mice, such as TNF receptor II(TNFRII), GITR and CD27 (Ellis and Braley-Mullen, 2015). However, the phenotypic differences do not appear to correlate with differences in function (Ellis and Braley-Mullen, 2015).

Interestingly, the influence of B cells on Tregs appears to be temporal, since adding B cells to co-cultures of Tregs and effector T cells (Teff) prior to Treg/Teff interaction induces Treg activation. Conversely, adding B cells after Treg/Teff interaction has no effect (Braley-Mullen and Yu, 2000; Yu et al., 2012). Thus, B cells intervene with the Treg/Teff stimulation during a restricted window of time before the interaction between Treg and Teff has started.

### 4.2 Distinguished effects of B cells on Tregs

As immune regulatory cells, Tregs suppress cytotoxic CD8^+^ T cells, CD4^+^ T cells and B cells (Hori et al., 2003). Counterintuitively, these targets act on Treg cells. CD4^+^ T cell-derived IL-2 is necessary for the expansion of Tregs (Bayer et al., 2009). Also, membrane-bound TNF-α from activated cytotoxic CD8^+^ T cells ligate to corresponding receptors on the Tregs (Joedicke et al., 2014). B cells play essential roles in the early development of Tregs. The development of Tregs involves two stages. First of all, the interaction of TCR-MHC II-peptide and CD28^−^CD80/CD86 results in the production of thymic CD25^+^Foxp3^-^ pre-Tregs. Then, pre-Tregs advance into mature CD25^+^Foxp3^+^ Tregs *via* an IL-2-dependent process (Figure 1) (Lio and Hsieh, 2008). The thymus contains a low frequency of B cells in the medulla which express more CD80, CD86 and MHC II than splenic B cells (Lu et al., 2015) (Figure 1). B cells in the thymus function like APC cells. Their BCRs capture autoantigens and trigger the negative selection of auto-reactive thymocytes by eliminating auto-reactive thymocytes (Lu et al., 2015). Thymic B cells act during the first stage of Treg development, from thymic CD4^+^ T cells to pre-Treg cells, but do not aid in the development of pre-Tregs into mature Tregs (Lu et al., 2015).

**FIGURE 4:**
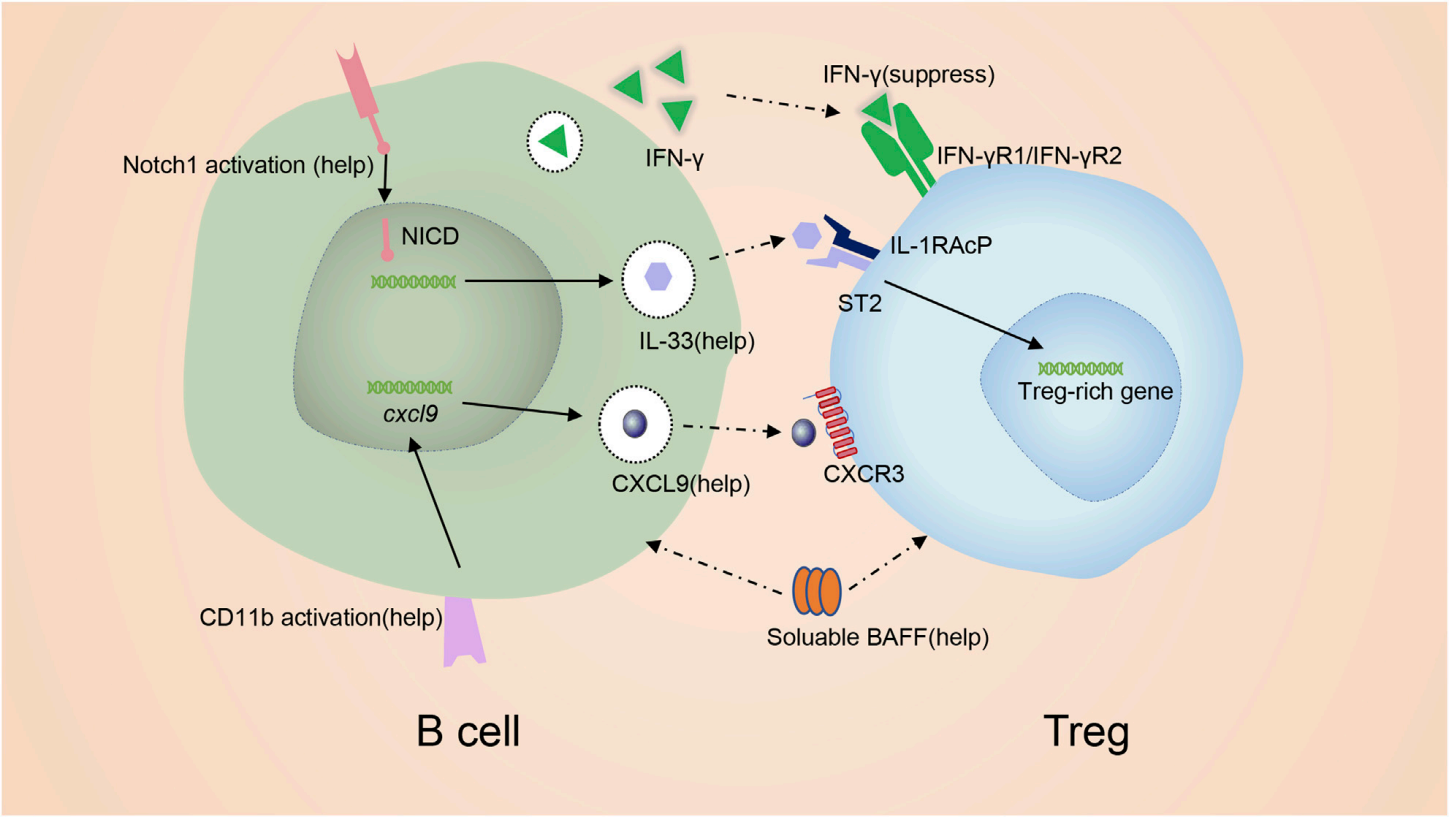
mechanism of promoting Treg by B cells. IFN-γ from B cells inhibits Tregs. GITR-L on B cells maintains Tregs at a sufficient level. Notch signaling induces the expression of IL-33. IL-33 binding to ST2 to favor the production of Treg. CD11b signal in B cells induce the production of CXCL9 which is capable of recruiting Tregs. BAFF can also promote Tregs.

Next, we carefully summarize the mechanisms of B cells acting on Tregs. B cells are able to inhibit Tregs *via* interferon-gamma (IFN-γ). In mice with experimental arthritis, interferon-gamma (IFN-γ) from B cells is found to inhibit Tregs (Ellis and Braley-Mullen, 2017) (Figure 4), and the absence of B-cell-derived IFN-γ facilitates the transformation of CD4^+^ T cells into Tregs (Olalekan et al., 2015). GITR ligand (GITR-L), IL-33, Chemokine (C-X-C motif) ligand 9(CXCL9) and B cell activating factor (BAFF) from B cells exert helpful effects on Tregs (Figure 4). Reports demonstrated that GITR-L on B cells maintained Tregs at a sufficient level to inhibit EAE (Ray et al., 2012). In mice with overexpression of GITR-L in B cells, the population of Tregs is remarkably increased (van Olffen et al., 2009). Besides, in mice with EAE who received anti-CD20 IgG1, researchers have identified a new B cell subset BD_L_, which only expressed low levels of IgD (Ray et al., 2019). BD_L_ cells functioned as regulatory cells and were involved in the recovery from EAE by inducing Treg proliferation and maintaining Treg homeostasis *via* the expression of GITR-L^121^. Thus, the GITR-L signal positively regulates Tregs. However, some research drew the opposite conclusion, in which GITR-L signaling in B cells blocked the expansion and function of Tregs during allograft transplantation (Olson et al., 2004; Ephrem et al., 2013; Nowakowska and Kissler, 2016). This phenomenon can be partly explained by that some Tregs induced during allograft transplantation are pTregs rather than tTregs (Olson et al., 2004; Ephrem et al., 2013; Nowakowska and Kissler, 2016). Interestingly, when B cells are cultured with Tregs in a 1:1 ratio, blocking GlTR-L can partially inhibit B cells from prompting Treg expansion (Moore et al., 2017). When B cells are cultured with Tregs at a 10:1 B cell to Treg ratio, similar to the physiological distribution, inhibition of GlTR-L does not typically change the proliferation of Tregs (Moore et al., 2017). Mature B cells with dysregulated Notch1-activation are associated with increased numbers of Tregs and Th2 cell-related cytokines in an IL-33-dependent manner (Arima et al., 2018) (Figure 4). Activation of Notch signaling positively correlates with the expression of IL-33 ^125^. Meanwhile, ST2, the IL-33 receptor, is expressed by Tregs. IL-33 binds to ST2 of Tregs to increase the production of Treg–associated cytokines, strengthen TGF-β-mediated differentiation of Tregs and provide an indispensable signal for Treg maintenance in inflammatory tissues (Schmitz et al., 2005; Schiering et al., 2014; Han et al., 2015; Arima et al., 2018). And blocking IL-33 abrogates Treg responses triggered by B cells. CD11b^+^ B cells in PPs during colitis are capable of recruiting Tregs into PPs *via* the production of CXCL9 (Wang et al., 2018a) (Figure 4). B cell activating factor (BAFF), belonging to the TNF superfamily, is known for enhancing B cell survival and differentiation. Interestingly, BAFF can also promote Tregs (Figure 4) (Walters et al., 2009). The frequency of Tregs is increased in mice overexpressing BAFF (B6. BTg) compared to WT mice. As discussed above, in μMT mice, the number of Tregs decreased due to the deficiency of B cells (Moore et al., 2017). However, the increased Treg response is absent when irradiated B6. BTg mice are given bone marrow from μMT mice (Walters et al., 2009). Thus, it is difficult to determine whether BAFF augments Tregs in B6. BTg mice or not. Yu et al. showed that both BAFF and B cells contributed to Tregs numbers *in vivo*. The contribution of B cells does not depend on the presence of BAFF, while the contribution of BAFF is strictly B cell dependent (Stohl and Yu, 2020).

## 5 Regulatory B cells

### 5.1 Introduction of regulatory B cells

Bregs is actually a term to describe B cells with regulatory functions (Mauri and Bosma, 2012). They are rare but have an essential role in maintaining immune tolerance (Mauri and Bosma, 2012). IL-10 is the defining hallmark of Bregs. But as an intracellular factor, IL-10 is not an adequate marker for identification (Mauri and Bosma, 2012). In fact, mouse and human Bregs lack reliable surface markers or lineage-limited biomarkers comparable with Foxp3 in Tregs, thus the immune regulation of Bregs remains unclear. Nowadays, eGFP reporter mice, which express eGFP under the control of the IL-10 promoter, is used to identify IL-10-producing Bregs (Rosser et al., 2014; Piper et al., 2019). Some scholars suggest that Bregs may be in a responsive state, and all B cells have the potential to produce IL-10 under certain environments (Shen et al., 2014). Different Breg subsets been comprehensively reviewed by wang et al. (Wang et al., 2020a).

Bregs have been widely identified as key regulators of autoimmunity, tumors and infection (Iwata et al., 2011; Mohammed et al., 2013; Wang et al., 2014; Woo et al., 2014; Wang et al., 2020a). The role of Bregs in autoimmunity has nothing to do with autoantibody production but is strongly associated with cytokine secretion such as IL-35, IL-10 and TGF-β (Wang et al., 2014). Mice with IL-10-deficient B cells form severe EAE, and the symptoms are alleviated through adoptive transfer of IL-10-generating B cells from WT (Fillatreau et al., 2002). The anomalous augmentation of Bregs inhibits immune responses *via* impairing T-cell responses (Choi et al., 2017). Bregs have been found in both hematologic tumors and various solid tumors (Iwata et al., 2011; Mohammed et al., 2013; Woo et al., 2014). They promote tumor growth *via* interactions with multiple immune cells within the carcinoma microenvironment, such as tumor-associated macrophagocytes, CD4^+^ and CD8^+^ T cells, natural killer (NK) cells, and myeloid-derived suppressor cells (MDSC). Moreover, Bregs can directly interact with tumor cells (Iwata et al., 2011). In mice with *Babesia microti* infection, the proportion of Bregs and Tregs are elevated and adoptive transfer of *Babesia microti* infection induced Bregs to recipient mice increase the susceptibility to *Babesia microti* infection (Wang et al., 2020a).

### 5.2 The differentiation of regulatory B cells

The induction of Brges has been widely discussed. Cell: cell contacts involving BCR signal and CD40^−^CD40L interaction, and cytokines like BAFF, IFN-α, IL-35, IL-1β, IL-21, and IL-6 are needed for the expression of IL-10 and development of Bregs. Earlier studies demonstrated that BCR engaging with lipopolysaccharide induces B cells to express IL-10, suggesting that BCR signal played an essential part in the proliferation of Bregs (Tian et al., 2001). This process is related to Aryl hydrocarbon receptor (AhR), which is highly expressed in IL-10^+^ Bregs compared with IL-10^-^ regular B cells (Piper et al., 2019). Intracellularly, AhR binds to transcriptional start sites (TSS) of the *ll-10* locus in Bregs (Piper et al., 2019). Plasmacytoid dendritic cells (pDCs) induce the expansion of Bregs from immature B cells *via* IFN-α and CD40^−^CD40L stimulation (Yoshizaki et al., 2012; Menon et al., 2016). Interestingly, Bregs, in turn, suppress IFN-α from pDCs *via* lL-10 and inhibit pDC activation, thus forming regulatory feedback between Bregs and pDCs (Menon et al., 2016). BAFF, binding to the receptor in B cells to activate AP-1, can transmit a positive signal to the induction of the Breg in marginal zone regions (Yang et al., 2010).

IL-35 belongs to the IL-12 family and consists of an α subunit IL-12p35 and a β subunit Ebi3. IL-35 binds to the IL-35 receptor on B cells to promote the differentiation of Bregs through stimulating STAT1/STAT3 (Wang et al., 2014). STAT3 is associated with the production of IL-10. Treatment with RSV, a powerful depressor of phosphorylated STAT3, successfully decreased the formation of pSTAT3 and prevented the generation of Bregs (Lee-Chang et al., 2013). Mice deficient in IL-35 or defective in IL-35-signaling show fewer Bregs than wild-type mice (Shen et al., 2014; Wang et al., 2014; Kang et al., 2020). Interestingly, Bregs generate IL-35 as well. The pairing of IL-12p35 and Ebi3 is not the necessary condition for IL-35 to promote Bregs. Egwuagu et al. demonstrated that only subunit IL-12p35 or only subunit Ebi3 could also exert some of the immune-regulatory properties of IL-35 (Dambuza et al., 2017; Ma et al., 2019).

Notably, Gut microbiota has a distinctive role in promoting Bregs. Rosser et al. demonstrated that the percentage and number of Bregs were promoted by gut microbiota in the spleen and mesenteric lymph nodes in an IL-1β and IL-6 dependent manners (Shaw et al., 2012; Rosser et al., 2014). Additionally, Mishima et al. showed that enteric microbiota preferentially induced Bregs in the intestine through TLR2/MYD88/PI3K pathway (Mishima et al., 2019). *In vitro*, the treatment of bacteria-derived oligodeoxynucleotides(CpG) transforms human B cells in buffy coats into Breg-like cells (Gallego-Valle et al., 2018). The Breg-like cells own elevated Breg-related markers, like IL-10, CD71 and PD-1, and exert suppressive functions (Gallego-Valle et al., 2018). Female MRL/lpr mice treated with DNA from the gut microbiota show elevated Bregs in both the mesenteric lymph node and spleen (Mu et al., 2020).

IL-21 from Tfh cells is important for inducing the expression of IL-10 in Bregs, as the supernatants from cultured Tfh cells including IL-21 induce the IL-10 production and treatment of anti-IL-21 antibodies blocks this effect (Yang et al., 2014). Also, in mice with multiple sclerosis, Yoshizaki et al. verified the maturation of Bregs required signals from IL-21 (Yoshizaki et al., 2012). The positive role of IL-21 on Brges may also associate with activated phosphorylated STAT3 like IL-35 (Yang et al., 2014). Besides, Tfh cells also elevate the inhibitory function of Bregs (Wang et al., 2018b). From another point of view, Tfh cells, as pro-inflammatory cells, induce the proliferation of anti-inflammatory Bregs in an IL-21-dependent manner, indicating regulatory feedback in GCs to reduce excessive inflammation responses.

The expression of transcription factors like Blimp-1 and Hypoxia-inducible factors (HIFs) is enriched in Bregs. Blimp-1 alone represses IL-10, while Blimp-1 with activated STAT3 upregulates IL-10 (Wang et al., 2019). Besides, HIFs, as critical transcription factors mediating the metabolism and functions of immune cells, control the expression of IL-10 in Bregs (Meng et al., 2018).

### 5.3 Mechanism of inducing regulatory T cells by regulatory B cells

Bregs can induce the expansion of Tregs (Figure 1) and inhibit Th17 cells (Wang et al., 2014; Lu et al., 2017; Tarique et al., 2018; Gao et al., 2019). They can strengthen the expression of CTLA-4 on Tregs (Kessel et al., 2012). When Peripheral Blood Mononuclear Cells (PBMCs) are co-cultured with CD4^+^ T cells from human beings, the CD4^+^ T cells synthesize fewer IFN-γ and IL-17 but more IL-4 and then convert into Tregs (Liu et al., 2016). And after depleting Bregs from PBMCs, the number of Tregs and the expression of CTLA-4, TGF-β and IL-10 significantly decreased (Liu et al., 2016). Tregs, as anti-inflammatory cells, are responsible for maintaining self-tolerance and suppressing immune responses, while proinflammatory Th17 cells help induce and accelerate inflammation. The shifting of Th17/Treg balance toward Th17 can be observed in various autoimmune diseases, such as SLE, RA, multiple sclerosis (MS), psoriatic arthritis (PsA), ankylosing spondylitis (AS), uveitis and Crohn’s disease (CD) and inflammatory bowel disease (IBD) (Beringer et al., 2016; Dambuza et al., 2017; Fasching et al., 2017). In these diseases, Bregs show the potential to exert immunosuppressive effects by increasing the percentage and amount of Tregs and attenuating the percentage and amount of Th17 cells (Hong et al., 2019), and in Sarcoidosis model induced by Propionibacterium acnes (PA), decreased Bregs were associated with aggravated inflammation (Mengmeng et al., 2020).

As we mentioned before, regular B cells also have the ability to promote Tregs (Figure 1). Thus, it is important to determine the difference between regular B cell-mediated promotion and Bregs-mediated promotion. Research demonstrated that CD4^+^ T cells co-cultured with IL-10-deficient B cells generated more Tregs compared with CD4^+^ T cells alone, however, the increase was smaller than CD4^+^ T cells co-cultured with IL-10-sufficient Bregs, which indicated that Bregs showed a more powerful role in promoting the proliferation of Tregs compared with regular B cells (Gao et al., 2019). Although, when B cells are cultured with Tregs or regular CD4^+^ T cells, the proportion of Bregs has no obvious change (Gao et al., 2019).

**FIGURE 5:**
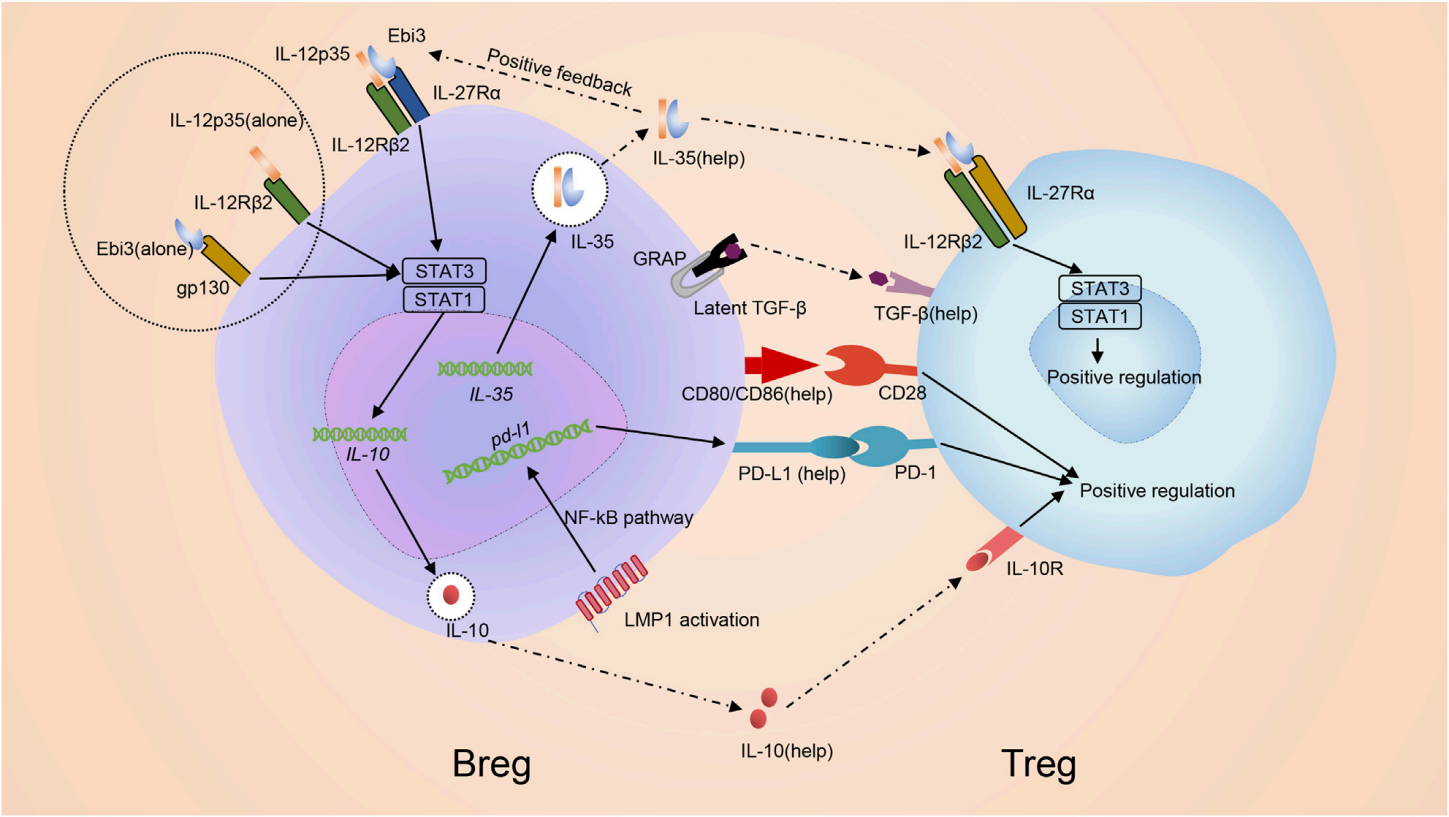
Mechanism of inducing regulatory T cells by regulatory B cells. Bregs promote development of Tregs via both cell: cell contact with CD4^+^ T cells and the secretion of immunomodulatory cytokines. IL-35 promotes the differentiation of Bregs through stimulating STAT1/STAT3 via binding pairing of IL-35 receptor IL-12p35 and Ebi3 or binding subunit IL-12p35 and Ebi3 alone. Bregs themselves can produce IL-35 and promote Tregs in an IL-35-mediated manner. IL-35 receptor in Treg, which is composed of an IL-12Rβ2/gp130 heterodimer, is activated to induce STAT1-and STAT4-signaling to benefit Tregs. Besides, Bregs can also secret other immunoregulatory cytokines, in particular IL-10 together with TGF-β, to regulate Treg. Additionally, co-stimulatory molecules CD80/CD86 are also critical for Bregs to promote development of Tregs. Breg can generate PD-L1 due to the MAPK/NF-κB pathway by LMP1 and advance the growth of Tregs *via* PD-L1/PD-1 signaling.

Bregs promote Tregs *via* cell: cell contact and secretion of immunomodulatory cytokines (Figure 5). The role of Breg-CD4^+^ T cell interaction is determined using transwell chambers which provide a physical barrier between cells but allow cytokines to pass through (Hong et al., 2019). The transwell chamber only partially prevents the proliferation of Tregs, indicating that both cell: cell contact and cytokines are involved in regulating Tregs by Bregs (Hong et al., 2019). Bregs produce various immunoregulatory cytokines like IL-35 and TGF-β to modify the response of T cells, especially Treg development (Kessel et al., 2012; Mauri and Bosma, 2012; Mielle et al., 2018). Additionally, co-stimulatory molecules CD80/CD86 are also critical for Bregs to promote the development of Tregs (Mielle et al., 2018) (Figure 5). TGF-β, in combination with IL-2, is initially required for Treg development by inducing transcription of FoxP3 (Morikawa et al., 2016). Experimental data showed that after adding TGF-βsRII, the TGF-β blocker, to the co-culture medium involving CD4^+^ T cells and Bregs, the amount and proportion of Tregs decreased. When adding IL-10sRα and TGF-βsRII to the co-culture involving CD4^+^ T cells and Bregs, the amount and proportion of Tregs were similar to adding TGF-βsRII alone, indicating TGF-β played a leading role in modifying Breg-related upregulation of Tregs (Figure 5) and IL-10 might not involve in this process (Kessel et al., 2012; Hong et al., 2019). Bregs are able to promote Tregs in an IL-35-mediated manner (Figure 5). The IL-35 receptor in Treg is a heterodimer consisting of IL-12Rβ2 and gp130, and can be activated to induce STAT1-and STAT4-signaling to benefit Tregs (Collison et al., 2007). It is observed from uveitis mouse models that exogenic mouse recombinant IL-12p35 combined with IL-12Rp2 to enhance Tregs (Dambuza et al., 2017). Besides, IL-35 signaling is required for the maximum function of Tregs (Huang et al., 2017). Hence, combined with the previous conclusion, IL-35 provides stimulation to both Tregs and Bregs (Collison et al., 2007; Collison et al., 2010).

PD-L1/PD-1 signaling may help Bregs promote Tregs. When Epstein-barr virus (EBV) infects B cells, EBV latent genes are transcribed and drive infected B cell perpetuation by dysregulating key activation pathways, forming a new B cell subset named as EBV latency III–transformed B cells (Auclair et al., 2019). Like Bregs, EBV latency III–transformed B cells secrete IL-10, TGF-β and IL-35 (Auclair et al., 2019). Thus, EBV-infected B cells are proposed to be a subset of Bregs. These cells generate PD-L1 due to the MAPK/NF-κB pathway by Latent Membrane Protein 1 (LMP1), a protein encoded by the main EBV oncogenes. EBV latency III–transformed B cells advance the growth of Tregs *via* PD-L1/PD-1 signaling (Auclair et al., 2019) (Figure 5). The results help clarify why EBV-related Diffuse large B-cell lymphomas (DLBCLs) arise in a more fragile context since the total population of Tregs in these patients increase and the immunosuppressive function enhances accordingly (Chang et al., 2015).

Conversely, experiments confirmed that Tregs have no functions in the maintenance and generation of Bregs (Gao et al., 2019). Gong et al. showed that the deficiency of Tregs in the PBMCs from human beings did not alter the frequency of Bregs and their function of producing IL-10 (Gong et al., 2015).

## 6 Treg-of- B cells

### 6.1 The introduction of Treg-of-B cells

**FIGURE 6:**
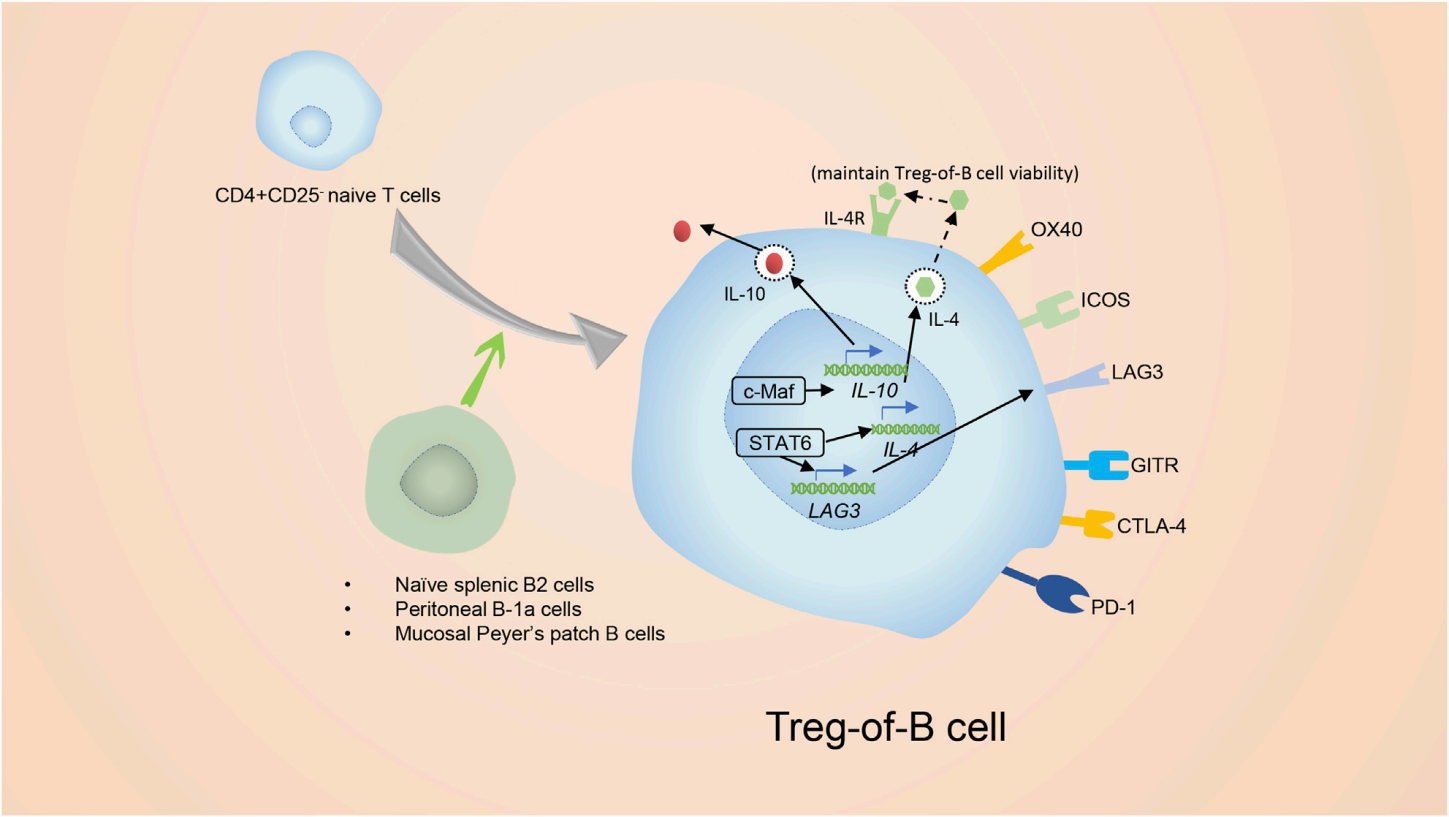
The introduction of Treg-of-B cells. Splenic B2 cells and mucosal Peyer’s patch B cells as well as peritoneal B-1a cells are able to promote the conversion of CD4^+^ CD25^−^ T cells to CD4^+^ Foxp3^-^ Tregs. The CD4^+^ Foxp3^-^ Tregs are known as Treg-of- B cells. Treg-of- B cells express molecules similar to conventional Tregs, for example ICOS, LAG3, GITR and OX40. In addition, Treg-of- B cells express CTLA4 and PD-1 as well, but do not express Foxp3. Activation of STAT6 was essential for the development of Treg-of-B cells. Phosphorylated STAT6 elevates the expression of LAG3, which is involved in generating and maintaining the function of Treg-of-B cells. After culture with B cells, Treg-of-B cells elevate their level of c-Maf which regulates expression of IL-10, ICOS, and CTLA4.

Treg-of-B cells are a special subset of Tregs that lack FoxP3 but still have inhibitory features and are induced by naïve B cells (Xie et al., 2015). Naïve splenic B2 cells and mucosal Peyer’s patch B cells as well as peritoneal B-1a cells are able to induce the conversion of CD4^+^ T cells to CD4^+^ Foxp3^-^ Treg-of-B cells without the additional cytokines or molecules (Figure 6) (Xie et al., 2015). Treg-of- B cells express molecules similar to conventional Tregs, such as IL-10, ICOS, LAG3, GITR, OX40(CD134), CTLA4 and PD-1, but surprisingly do not express FoxP3 (Chu and Chiang, 2012; Hsu et al., 2015; Chien et al., 2017).

### 6.2 The proliferation mechanism of Treg-of-B cells

Inducing Treg-of-B cells depends on cell: cell contact and is independent of IL-10 (Chu and Chiang, 2012; Hsu et al., 2015). According to a study on the Peyer’s patch, activation of STAT6 was essential for the development of Treg-of-B cells (Chu et al., 2020) (Figure 6). Phosphorylated STAT6 elevates the expression of Lymphocyte Activation Gene 3 (LAG3), which is involved in generating Treg-of-B cells and maintaining their capacities, and anti-LAG3 antibody treatment abolishes the inhibitory function of Treg-of-B cells (Chu and Chiang, 2015; Chen et al., 2016). Nevertheless, the upstream of phosphorylated STAT6 in Treg-of-B cells is not clear. IL-4 is widely known for inducing STAT6 phosphorylation, but in Treg-of-B cells, IL-4 is the downstream of STAT6 phosphorylation to maintain the viability but not the inducer, and it does not engage with the development of Treg-of-B cells (Chu et al., 2020). After constant stimulation with B cells, Treg-of-B cells show elevated c-Maf (a transcriptional factor that regulates the expression of IL-10, ICOS, and CTLA4), secrete more IL-10 and simultaneously increase the expression of CTLA4 and ICOS (Chien et al., 2017).

### 6.3 The capacity of Treg-of-B cells

In mouse models, Treg-of-B cells were shown to inhibit Th1 and Th17 from producing cytokines and inhibit the develpoment of effector T cells (Chu and Chiang, 2015; Chen et al., 2016). Both non-antigen-specific and antigen-specific Treg-of-B cells have inhibitory capacities, and the antigen-specific Treg-of-B cells also exert non-antigen-specific inhibitory functions (Chien et al., 2015).

The role of Treg-of-B cells has different manifestations among different diseases. They can alleviate the severity of rheumatoid arthritis, mitigate Th2 cell-related airway hypersensitivity, and down-regulate high titers of IgE (Reichardt et al., 2007; Chu and Chiang, 2012). In addition, Treg-of-B cells also repress the side effects of allogeneic heart transplants (Walters et al., 2009). Administration of Treg-of-B cells protects mice from T cell-triggered IBD (Shao et al., 2016a) and reduces the severity of joint injury in collagen-induced arthritis (Chen et al., 2016). Thus, Treg-of-B cells can serve as an economical strategy for T-helper-cell-mediated inflammatory diseases.

Treg-of-B cells can produce high levels of IL-10 (Shao et al., 2016b) which performe a partial role in Treg-of-B cell-mediated suppression. Chen et al. confirmed IL-10 is important for Treg-of-B cells to inhibit effector T cells (Chen et al., 2016), but Treg-of-B cells in IL-10-deficient mice also prevent the development of T cells *in vitro* and *in vivo* (Shao et al., 2016a). It is thought that IL-10 KO Treg-of-B cells boost non-IL-10 molecules to offset the lack of IL-10, however, data shows regulatory molecules did not change (Shao et al., 2016a). LAG3 and CTLA4 are also important for Treg-of-B cells to exert inhibitory effect (Chen et al., 2016; Chien et al., 2017). In mice with monosodium urate (MSU)-induced gout, Treg-of-B cells migrate into draining lymph nodes to mitigate arthritis through repressing NLR family pyrin domain containing 3(NLRP3)-related inflammation triggered by macrophages (Huang and Chiang, 2021). NLRP3 inflammasome is a multi-protein complex and its activation involves two steps. Step one is TLR stimulation which induces the transcription of pro-IL-1β *via* the NF-κB pathway. Step two is that Adenosine Triphosphate (ATP) or MSU crystals result in NLRP3 inflammasome assembly which then binds to caspase-1 to induce the delivery of IL-1β (Aizawa et al., 2020). Treg-of-B cells inhibit NF-kB pathways of macrophages *via* cell:cell contact, which culminates in the suppression of IL-1β generation from macrophages (Huang and Chiang, 2021). In general, the molecules mediating Treg-of-B cells, regulatory mechanisms remain to be investigated.

## 7 Interaction of B cells and regulatory T cells in different diseases

### 7.1 The effects of B cells on Treg in diseases

In tumors, B cells play a part of role by inducing Tregs, and understanding the mechanism may help improve the prognosis of certain tumor patients. The deficiency of B cells induces antitumor effects in mice with EL4 thymoma, MC38 colorectal cancer, EMT-6 mammary carcinoma (Shah et al., 2005; Tadmor et al., 2011; Zhang et al., 2013). In these tumor models, deletion of B cells leads advanced T cell development, Th1 cytokine generation, infiltration of NK cells, and cytolytic T cell responses. Especially in the EMT-6 mammary carcinoma, reduced B cells are associated with decreased proliferation of Tregs (Shah et al., 2005; Tadmor et al., 2011; Zhang et al., 2013). In the co-culture system of intertumoral ICOS^+^CD4^+^ T cells and ICOS^+^ Follicular Lymphomas B cells (FL B cells), the ICOS/ICOSL interaction induces the generation of Tregs (Le et al., 2016). The intertumoral Tregs comprises of about 15% intertumoral CD4^+^ T cells (Yang et al., 2007). This subset directly or indirectly suppresses FL B cell responses upon activation (Le et al., 2016). Additionally, CD70^+^ lymphoma B cells also exert remarkable roles in inducing the transcription of Foxp3 in intertumoral CD4^+^ T cells and the interaction of CD27/CD70 is involved in this process (Yang et al., 2007).

CD40L-stimulated B cells (CD40L-sBc) function as APCs *in vitro* to promote the development of CD4^+^ T cells, form patients with autoimmune diseases, to Treg (Bluestone et al., 2015). Alonso-Guallart et al. expanded CD40L-sBc derived from MHC-diverse macaques and used them to stimulate and expand Tregs. These expanded Tregs with poly specificities only secreted small amounts of inflammatory cytokines, inhibited naive T cell responses and could be activated by APCs with MHCs shared by the expanded CD40L-sBc (Alonso-Guallart et al., 2021). The new approach shows the potential in clinical applications (Alonso-Guallart et al., 2021).

### 7.2 The effect of Bregs on Tregs in cancers

Bregs have been widely identified as key regulators to tumors and the number and proportion of Bregs is elevated in various tumors. Wei et al. demonstrated that the proportion of ascitic Bregs were linked to a progressive clinical tumor phase (Wei et al., 2016). In HCV-related hepatocellular carcinoma (HCC), upregulated Bregs and Tregs are associated with HCV-related HCC progression (Hetta et al., 2020). Just like the positive effect of Bregs on Tregs as described above, the increased frequency of Bregs in tongue squamous cell carcinoma (TSCC), non-small cell lung cancer (NSCLC), and HCV-related HCC is linked to elevated Treg infiltration along with decreased survival time (Zhou et al., 2016; Liu et al., 2020).

The induction effect of Bregs to Tregs can be observed in many tumors. When CD4^+^ T cells are cultured with Bregs from acute myeloid leukemia (AML), Tregs are significantly increased (Shao et al., 2016b). In 4 T1 tumorous mice, Olkhanud et al. discovered unique tumor-evoked Bregs (tBregs), identified as CD19^+^CD25^+^ B220^+^ B cells (Zhang et al., 2013). When cultured with tBregs, CD4^+^ T cells elevate the transcription of FoxP3 and differentiate into Tregs in a TGF-β-dependent process (Zhang et al., 2013). Also, the adoptive transfer of tBregs into WT mice increases the proliferation of Tregs in the periphery (Zhang et al., 2013). Furthermore, in 4 T1 tumor mice that lack T and B cells, Treg-mediated metastasis in the lungs is enhanced after administration of tBregs and non-Tregs, implying that there is a tBreg-related induction of non-Tregs to Tregs (Olkhanud et al., 2011; Wejksza et al., 2013). These experiments suggest that tBregs are expanded in the tumor micro-environment, and in turn facilitate tumor metastasis *via* TGF-β-mediated differentiation of non-Tregs to Tregs. To prove this notion, Lee-Chang et al. showed that resveratrol (RSV), an inhibitor of phosphorylated STAT3, could restrain the expansion of tBregs and Tregs, and also repressed the enlargement of B16 and 4 T1 tumors (Lee-Chang et al., 2013). Altogether, they revealed that suppressing phosphorylated STAT3 in Bregs worked as an anti-tumor treatment (Yang et al., 2013; Ren et al., 2014). Among the entire chronic lymphocytic leukemia (CLL) cell compartment, B-CLL cells with high expression of CD38 are phenotypically similar to Bregs and termed as Breg–like CLL cells (DiLillo et al., 2013). Breg–like CLL cells secrete excessive levels of IL-10 and TGF-β, inducing the differentiation of naive T helper cells into Tregs (Manna et al., 2020). Both the induced Tregs and Breg–like CLL cells express CD38 and are eradicated *via* the treatment of anti-CD38 monoclonal antibody (Manna et al., 2020). Data showed that CD38^+^ Tregs occupied more than 50% of the Tregs in PBMCs in CLL patients (Manna et al., 2020). Hence, targeting CD38 may be a new treatment in CLL, which would result in the loss of both Tregs and Breg-like CLL cells to regulate the tumor micro-environment and promote reconstitution of the immune system (Manna et al., 2020).

### 7.3 Breg and its correlation with Treg in transplantation

Bregs also represent a vital regulator in transplantation. The survival of tissue allogeneic transplantation is enhanced in mice which are with ablation of bone marrow, then transplanted with skin allograft and then followed by syngeneic bone marrow transplantation (BMTx) (Gorczynski et al., 2018). Bregs are involved in this process by regulating the capacity of Tregs from donors (Alonso-Guallart et al., 2021). Studies demonstrated that the administration of Bregs in mice with post-BMTx prolonged survival of mice and elevated Treg levels (Gorczynski et al., 2018), and TGF-β aided in this process (Lee et al., 2014).

Both Bregs and naive B cells can induce Tregs, but their functions are different in transplantation models. Bregs induce more FoxP3 in CD4^+^ T cells compared to naive B cells (Lee et al., 2014). Administration of Bregs to grafted recipients confers more graft survival, but administration of naive B cells to recipients do not lengthen graft survival (Lee et al., 2014). Administration of Bregs elevates the amount of Tregs in recipient's spleens, while administration of naïve B cells or graft alone does not (Lee et al., 2014).

Data showed that Sirolimus alleviated steroid resistance in liver transplantation patients and induced long-term immune tolerance (Song et al., 2020). This is partly explained by that Sirolimus augments the number of Bregs and Tregs in liver transplant recipients and TGF-β1 and IL-10 are important in this process (Song et al., 2020). The increase of Tregs is partially suppressed when either IL-10 or TGF-β1 is inhibited and even vanished when both IL-10 and TGF-β1 are neutralized (Song et al., 2016).

### 7.4 Breg in the therapy of patients with autoimmune diseases

Bregs and Tregs also show essential roles in autoimmune diseases. The frequency of Bregs is significantly increased in PBMCs from patients with Crohn’s disease and the frequency of Tregs is significantly decreased (Lin et al., 2020). Upon infliximab treatment, the frequency of Tregs and Bregs increases, and the frequency of Breg is positively associated with the frequency of Treg all the time (Lin et al., 2020). It seems that remission of disease is always accompanied with increased frequency of Bregs and Tregs. Furthermore, ROC curve analysis shows that the detection of these two types of immune cells opens new windows for predicting the effect of infliximab in CD patients during an active phase (Lin et al., 2020).

Evidence showed helminth infection can lower the incidence and reduced the acceleration of allergic asthma (Gao et al., 2019). In murine model experiments, Li et al. demonstrated that infecting H. polygyrus could dramatically inhibit OVA-induced allergic airway inflammation (AAl) and induce substantial responses of Bregs and Tregs in the spleen and MLNs of the mice (Gao et al., 2019). Administration of both Bregs and Tregs can obviate lung immunopathology in mice with AAI (Gao et al., 2019).

### 7.5 Bregs and its correlation with Treg in inflammation and infection

The discussion about Bregs is not just in typical carcinoma and autoimmune diseases but has extended to a wider field, such as organ-restricted or whole-body inflammation. Reports showed that Bregs might suppress the development of inflammation and prevent inflammatory damage. Jin et al. established an osteoporosis model by ovariectomy (OVX) in mice and then detected the frequency of immune cells from the spleen (Wang et al., 2020b). Data showed that both the proportion of Bregs and Tregs decreased during the process of osteoporosis (Wang et al., 2020b). Adoptive transfer of Bregs to OVX mice prevents the advancement of osteoporosis in the alveolar bone (Wang et al., 2020b). In mice with Periodontitis, adoptive transfer of periodontopathogen-specific Bregs alleviates alveolar bone resorption through reducing periodontal osteoclastogenesis (Shi et al., 2020).

In patients with chronic hepatitis B(CHB), the number of Bregs is significantly elevated and is positively related to glutamic oxaloacetic transaminase (AST) and alanine aminotransferase (ALT). And during the CHB infection, Bregs suppress anti-virus responses *via* IL-10 (Li et al., 2021). In mice with *Babesia microti* infection, both the proportion of Bregs and Tregs are elevated and adoptive transfer of *Babesia microti* infection induced Bregs to recipient mice increases the susceptibility to *Babesia microti* infection (Wang et al., 2020a).

## 8 Conclusion

Tregs are a unique subpopulation of CD4^+^ T cells, accounting for only a small portion of T cell pool but playing an indispensable role in maintaining immune homeostasis and self-tolerance. B lymphocytes which originated from bone marrow HSC, own humoral immune functions. In this article, we provided multiple perspectives to discuss the interaction between B cells and Tregs.

On the one hand, Tregs can inhibit B cells in CTLA-4 and TGF-β-dependent manners. Besides, perforin, granzyme B, Fas-FasL and PD-1-PD-L1 interaction exert roles in Tregs-induced B cell apoptosis. Then, we introduce Tfr cells, a subset of Tregs residing in germinal center, and discuss the effects of Tfr cells on GC B cells. Apart from suppressing Tfh cells to indirectly act on B cells, Tfr cells also aim directly on B cells. In the beginning, we talk about the influence of Tfr on GC responses. Then, we delve into the mechanisms that Tfr cells regulate GC B cells. Tfr cells negatively influence GC B cells through CTLA-4, TGF-β and neuritin-dependent manners. Additionally, IL-10 plays roles in regulating CG B cells by Tfr and surprisedly exert positive functions. Through this review, we find there are lots of questions that remained to be elucidated. Mature Tfr cells are identified as CXCR5^hi^ PD-1^hi^ CD25^low^ Foxp3^+^ Bcl-6^hi^ Blimp1^low^ T cells (Botta et al., 2017; Ritvo et al., 2017). However, Tfr cells were first described as CD25^hi^, like regular Tregs and precursor Tfr cells. And many of the characteristics originally assigned to Tfr cells are probably due to the mixture of Tregs, precursor Tfr cells and Tfr cells. Thus, some research of Tfr cells needs update due to the newest classification of Tfr cells. Besides, how Tfr exquisitely influences the cell cycle, CSR, somatic hypermutations and affinity maturation of GC B cells remain to be clear. Next, we summarize the diseases involved in the modulation of follicular regulatory T cells and the effects on germinal centers and give a brief introduction to Tregs with chimeric antigen receptor (CAR)technology, providing some clues to bring] Treg to clinical treatment.

On the other hand, Tregs are regulated by B cells. As shown in Figure 4, IFN-γ from B cells inhibits Tregs. GITR-L, CXCL9 and BAFF in B cells exert positive function to Tregs. Abnormal activation of Notch1 signal in B cells induces the secretion of IL-33, and IL-33 bind the receptor in Tregs to induce the population of them. Bregs are a heterogeneous subpopulation of B cells with the secretion of IL-10 and are able to induce Tregs. We give Bregs a brief introduction and then highlight the mechanism of them inducing Tregs. As shown in Figure 5, Bregs promote Tregs *via* cell: cell contact and the secretion of immunomodulatory cytokines, like IL-35 and IL-10 together with TGF-β. Treg-of-B cells, a newly identified Treg induced by Naïve splenic B2 cells and mucosal Peyer’s patch B cells as well as peritoneal B-1a cells but without FoxP3, has drowned our attention. We reviewed the development mechanisms of Treg-of-B cells and their regulation to immune responses. At last, we discussed the role of Bregs in various diseases, such as cancers, transplantation and autoimmune diseases, hoping to find the potential value of aiming Bregs for treatment.
